# Crop Straw Resource Utilization as Pilot Policy in China: An Event History Analysis

**DOI:** 10.3390/ijerph20053939

**Published:** 2023-02-22

**Authors:** Wuliyasu Bai, Long Zhang, Liang Yan, Xinyi Wang, Zhiqiao Zhou

**Affiliations:** 1 School of Economics and Management, China University of Geosciences, Wuhan 430078, China; 2 School of Business, Xinyang Normal University, Xinyang 464000, China; 3 School of Land Science and Technology, China University of Geosciences (Beijing), Beijing 100083, China

**Keywords:** crop straw resource utilization, diffusion of innovation theory, pilot policy, event history analysis

## Abstract

Massively generated crop straw can be utilized and valorized with great economic and environmental benefits. The Chinese government has adopted the pilot policy of crop straw resource utilization (CSRU) for disposing of the straw and practicing waste valorization. This work took 164 counties in the Hebei Province of China as a case study, mapped the temporal and spatial characteristics of the diffusion of the CSRU pilot policy in this province, and conducted an Event History Analysis by establishing a binary logistic regression model to identify the specific factors that determine the diffusion of the CSRU pilot policy in China from the aspects of resource availability, internal capacity, and external pressure. It indicates that: (1) the CSRU pilot policy diffuses rapidly in Hebei Province, although it is still at the early stage of this policy diffusion; (2) the model explains 95.2% of the variance in adopting a pilot county, indicating the effectiveness of this model; (3) straw resource density has a positive impact on CSRU pilot selections, and it can increase the possibility of one county being selected as a CSRU pilot by 23.2%, while population density has shown a negative effect; (3) policy support from local government is a major internal factor that determines CSRU performance, and it can increase the possibility of one county being selected as a CSRU pilot nearly tenfold; proximity pressure from neighboring counties has a positive effect on the diffusion of the CSRU policy, and it also greatly increases the possibility of being selected as a CSRU pilot.

## 1. Introduction

Crop straw is generated massively in the agricultural sector, and traditionally, it was disposed of by directly burning in the open air, leading to various environmental issues, mainly atmospheric pollutions and greenhouse gas emissions [1,2]. To mitigate the negative impacts of open-air burning of crop straw on the environment, various pathways for utilizing crop straw are proposed and practiced, and they can be used as renewable resources for improving soil fertility, producing livestock feed, and generating renewable energy if used effectively [3]. In China, the effective utilization of crop straw can not only solve the tricky problems of disposing of crop straw, but also improve environmental quality, bring economic benefits, and create job opportunities, contributing to China’s Rural Revitalization Strategy [4]. As a large agricultural country, China produced more than 819 million tons of crop residuals in 2021, including the residuals of rice, wheat, corn, potato, cassava, peanut, rape, soybean, cotton, sugarcane, etc., and the straw yield is still growing slowly but steadily. Due to the policy of prohibiting burning crop straw in the open air since early this century, various pathways for realizing crop straw resource utilization (CSRU) have been proposed, and the ratio of CSRU has kept rising, even exceeding 90% in recent years. In fact, the National Development and Reform Commission and the Ministry of Agriculture of China compiled The Catalogue of Straw Resource Utilization Technology in 2014, which proposed five technological pathways for realizing CSRU in rural China [5]. With the proposal of carbon peak and neutralization, CSRU has become a major part of the national efforts in reducing carbon emissions and improving environmental sustainability [6].

To drive the reduction of carbon emissions and the diffusion of renewable energy technologies, various policies and measures, such as the banning order for open-air burning of crop straw, have been developed and implemented [7,8]. In fact, the present supporting policies in China mainly focus on technical processes, project subsidies, and the establishment of residual collection, storage, and transportation systems [4]. For instance, gasification is a promising technology that can be used for large-scale utilization of crop straw, so some scholars have focused on the policy that is aimed at promoting the commercialized utilization of crop straw with modern industrial technologies [9]. The subsidy policy has also been greatly emphasized in many studies. Sun et al. (2019) empirically examined the effect of the public policy on crop straw utilization in the case of the Jiangsu Province of China and stated that the present subsidy policy played an insignificant role as the subsidy was low and not directly delivered to farmers [7]. It is believed that the production operations, equipment purchase, storage, and transportation of CSRU should be established with the public subsidies [4]. To improve the efficiency of CSRU in China, the Chinese government has adopted a pilot policy of rewards and subsidies for CSRU.

**Figure 1:**
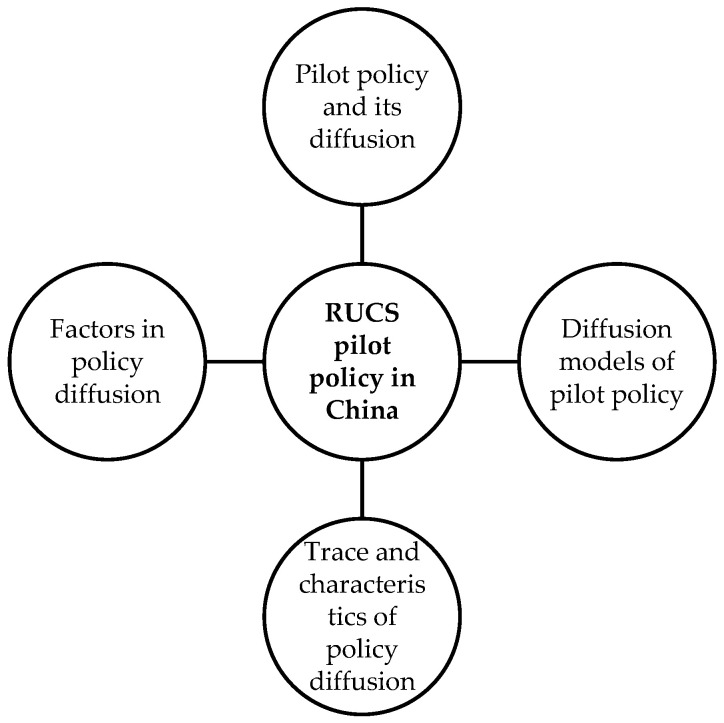
Streams of literature on the CSRU pilot policy.

Many studies have recently been conducted to investigate the CSRU pilot policy and its diffusions, and they can be summarized as the streams shown in Figure 1.

A pilot policy is like a form of experiment to “discover” new innovative objects, and then spread them to other places [10]. It is believed that the pilot works aim to mobilize and manage ambiguity and conflicts related to specific policy objectives, and explore new policy tools through policy innovation [11,12]. One purpose of a pilot policy is to generate practical, replicable, and scalable policy tools through the bottom-up governance model in the process of policy innovation [13], and different pilot policies have different influencing factors at different development stages [14]. Usually, the phenomenon of “pilot diffusion” can be explained by the diffusion of innovation theory (DIT) [15,16]. Walker (1969) believed that innovation diffusion is not to conceive or develop new ideas or plans. On the contrary, it is the condition in which decision makers are most likely to adopt new plans [17]. The diffusion of innovation policy refers to the development process in which the government knowledge at one department or level is applied to the governance of other departments or levels [18]. Policy innovation is the basis of policy diffusion, and the objects of policy diffusion are innovative policies or projects [19]. The exchange and interaction of innovation policies are emphasized, and policy innovation plays a significant role in the exchange activities [20,21].

From the numerous model construction studies, four basic diffusion models are summarized: national interaction model, regional diffusion model, leadership follow-up model, and vertical influence model [22,23,24]. Presently, the research on the diffusion of energy policy is more focused on internal policy diffusion, with the country as the basic unit [22,23,24], and different political systems and culture lead to different influencing mechanisms of policy diffusion [23]. Based on different policy cases in China, scholars have put forward the diffusion of innovation theory with Chinese characteristics. For instance, Zhang and Xiao (2020) believed that China’s policies are vertically diffused from top to bottom, mainly in the form of experimental governance [25]. The CSRU policy diffusion model in China conforms to the top-down diffusion characteristic, which is also the further excavation of the diffusion policy theory.

The trace of policy diffusion can be analyzed to identify the spatiotemporal distribution characteristics of the diffusion process. The S-shaped curve of the diffusion trace with “slow start-up period–accelerated mature period–slow and stable period” is recognized as the most common spatiotemporal distribution characteristic of policy diffusion [26]. In addition, three other characteristics of policy diffusion have also been identified: R-shaped, steep S-shaped, and ladder-shaped [27,28]. In terms of spatial distribution, government departments are more inclined to learn the advanced experience and imitate the successful policies of the adjacent areas due to the similarity of environmental conditions and availability of resources and experience [19]. Due to the influence of geographical and weather conditions on crops planting, some geographical characteristics and regional similarities can be witnessed, which may impact on the spatial distribution of CSRU policy diffusion within a region. Therefore, it is necessary to analyze the spatiotemporal distribution of the diffusion effects of the CSRU pilot policy.

In addition, scholars have paid great attention to the investigation of the influencing factors in the process of policy diffusion [29,30]. In fact, plenty of factors have been involved in policy diffusion, and most of them are multi-dimensional and comprehensive elements. Weiner (2002) summarized the influencing factors of policy diffusion as characteristic of innovation, innovators, and environmental background, which has been widely recognized by scholars [31]. Some other factors are also thought to have impacts on the diffusion of policy. For example, Walker (1969) proposed that economic strength has a significant impact on policy diffusion [17]; Volden (2006) believed that policy diffusion was closely related to the characteristics of states, population distribution, and financial situation [32]. Abel (2019) proposed that, in addition to geographical proximity and party channels, cross-regional networks are also a predictor of the diffusion process of climate policies [24]; Dagar et al. investigated the variations in technical efficiency of farmers with distinct land size across agro-climatic zones, implying the possible effect of difference in land size and agro-climatic zone on the adoption of CSRU [33]. The different institutional basis of intergovernmental relations between China and Western countries is also believed to result in the obviously different influencing factors and specific paths of policy diffusion [19]. Zhang and Xiao (2020) proposed that the pilot diffusion of innovative cities in the Chinese context would be affected by the experimental environment, basic conditions, and pilot proportion [25]. Li and Gu (2020) found that, in a pressure government system similar to the Chinese governance, the adoption of local government policies should be based on the implementation of superior orders on the one hand, and horizontal competition and response to people’s needs on the other hand [34]. Therefore, in the study of policy diffusion in China, in addition to considering the internal environment and its own resource conditions, the relationship between vertical management institutions and horizontal competition institutions should also be considered.

The pilot policy for CSRU promotion in China has been intensively discussed by scholars. For instance, He et al. (2018) analyzed the individual factors of farmers affecting their willingness to accept energy utilization of crop straw by using nonparametric estimation results [35]; Zhang et al. (2019) analyzed the problems existing in the implementation of the pilot project of CSRU in Huaibin County of China and put forward corresponding solutions [36]; Wang et al. (2022) discussed the hot areas, objects, and methods of CSRU pilot policy in China through text analysis [4]; Li et al. (2022), and Del Valle and Jiang (2022) summarized the factors affecting farmers’ willingness and behavior in comprehensive straw utilization [37,38]. Despite this, very few studies have been conducted to discuss the diffusion of the CSRU pilot policy. In fact, the diffusion effect of the pilot policy is particularly important for achieving and improving CSRU.

Thus, this study tries to answer the following questions:

- During the diffusion process of the CSRU pilot policy, what are the temporal and spatial characteristics of the current diffusion effect?
- What elements and factors should be considered when selecting or formulating the pilots to achieve the diffusion effect?
- How can we use the diffusion effect of the pilot policy to improve the CSRU efficiency?

The objective of this study is to explore the potential factors that may affect the diffusion process of the CSRU policy in China and identify the diffusion effects in the CSRU policy. The potential contribution of this study lies in the following points: (1) it tries to establish the pilot policy diffusion model of CSRU in China; (2) it summarizes the factors affecting CSRU policy diffusion from three aspects; (3) the effects of these influencing factors are tested to identify the important ones on CSRU policy diffusion.

The rest of this work is organized as follows: Section 2 presents the introduction and evolution of the CSRU pilot policy in China; Section 3 summarizes the various factors that affect the adoption and diffusion of the CSRU pilot policy in China and develops the research hypothesis; Section 4 describes the research sample, variable measurement, and model specification; Section 5 depicts the results of this study; and, finally, the conclusions and discussions are presented in Section 6.

## 2. Pilot Policies for CSRU Promotion in China

Crop straw was utilized as the major energy source for cooking and heating in rural China up until the 1980s [39]. However, with the improvement of rural living conditions, the massively generated crop straw has gradually been abandoned and directly burned in the fields. Due to the rapidly deteriorating air environment, the improper disposal of crop straw has been brought to the public’s attention, and the burning of crop straw in the open air has been prohibited nationwide. Instead, more attention has been paid to the utilization and valorization of rural crop straw.

According to China’s policy and practice in CSRU promotion, this paper summarizes the introduction and evolution of the CSRU pilot policy in China with three stages. (1) Project experiment and demonstration stage. In 2009, the Ministry of Agriculture and Rural Affairs (MARA) of China launched 16 large- and medium-sized pilot biogas projects with crop straw as the base material in 14 provinces, which initiated the large-scale promotion of CSRU in China. (2) Provincial pilot stage. In 2011, the National Development and Reform Commission (NDRC), MARA and Ministry of Finance (MOF) of China jointly issued the Implementation Plan for CSRU during the 12th Five Year Plan, which proposed the implement of the CSRU pilot policy and technology. In 2016, the MARA and MOF issued the Notice on Carrying out CSRU Pilot Policy to Promote the Improvement of Cultivated Land Quality, and invested CNY one billion to select ten provinces and autonomous regions as pilots for practicing CSRU, which is the first time the CSRU pilot policy was put forward at the central government level in China. (3) County pilot stage. Since 2017, the CSRU promotion has been implemented at the county level, and various supporting policies and efforts have been introduced intensively in the following years, as presented in Table 1. Since then, the county has been acknowledged as the basic unit for implementing the pilot policy of CSRU promotion. Apparently, the Chinese government is increasing its efforts in implementing the CSRU pilot policy, and the ultimate goal is to improve the utilization of rural solid waste and build a green and low-carbon environment in rural areas.

**Figure 2:**
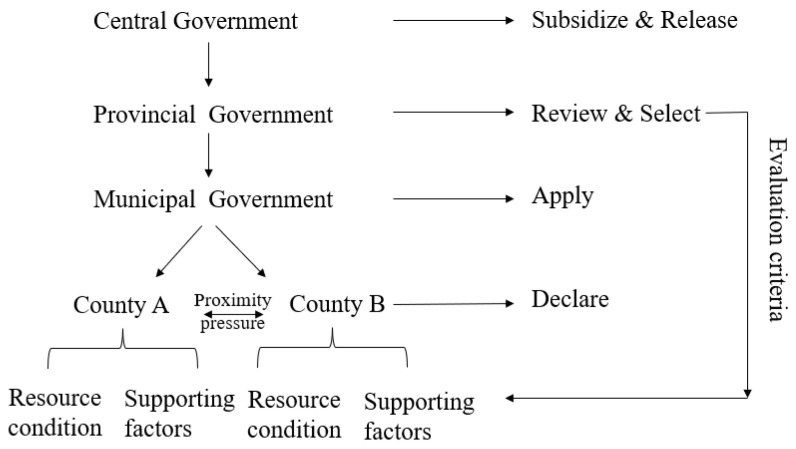
Procedure of CSRU pilot counties selection.

Presently, China uses the CSRU pilot policy of substituting subsidies with rewards. Under this subsidy policy, the local governments, mainly county and district governments, play a critical role in the last cycle in the implementation of CSRU, as they are the basic units of the subsidy distribution. The pilot selection process of the Chinese government includes initiative application of local government, national assessment and approval, etc., which reflects the combination of the central guidance and local initiative experiments [25]. Under this CSRU pilot policy with the form of replacing subsidies with rewards, the MARA and MOF of China take the lead in providing the financial subsidy funds. After the procedures of a county-level declaration, municipal recommendation, and review, the provincial governments determine the lists of CSRU pilot counties and submit them to the MARA. The approved pilot counties and districts take the lead in using the subsidy funds granted by the MOF to support a number of market players to develop CSRU projects. In practice, county governments are encouraged to submit pilot applications, then the municipal governments transfer the application to the provincial government, who determines the list of selected pilot counties after careful evaluation, mainly supporting areas with large amounts of crop straw, the heavy task of prohibition of burning, and great CSRU potentials. Finally, this list is provided to the MOF and MARA, who determine the final list of CSRU pilot counties and arrange the financial support. This whole schedule is shown in Figure 2.

By doing this, the Chinese government is aiming to promote the CSRU policy gradually by selecting a number of counties as pilots, which is called the CSRU pilot policy. By putting forward and implementing this pilot policy with rewards and subsidies for CSRU, China is aiming to establish a green and low-carbon oriented mechanism for realizing ecological utilization of crop straw.

Now it is still at the promotion stage of regional planning in CSRU promotion under the background of the Rural Revitalization Strategy [40], and the Chinese government has adopted the pilot policy for promoting CSRU, which aims to improve CSRU efficiency and the quality of China’s rural ecological environment by using the diffusion effect of policy innovation. Apparently, China has adopted a set of policies for promoting CSRU concentrated on pilots and demonstration. By clarifying the trace and identifying the characteristics of the diffusion of the CSRU pilot policy, this study can help decision-makers to formulate better policies to facilitate CSRU and improve CSRU efficiency around the country.

## 3. Factors Analysis and Research Hypotheses

**Figure 3:**
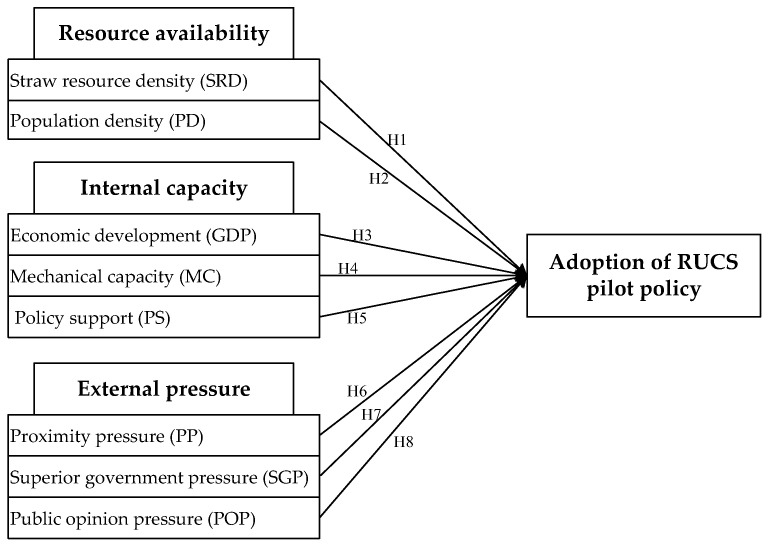
Relationship model of this study.

According to the characteristics of pilot selection, this paper summarizes the factors influencing CSRU pilot selection and policy diffusion by referring to the diffusion of innovation theory and China’s government policy mechanism from three aspects: resource availability, internal capacity, and external pressure, as shown in Figure 3.

### 3.1. Resource Availability

Resource availability influences the chance of policy adoption within a region [41,42]. Slack resources can allow being able to afford to purchase innovations, absorb failure, bear the costs of instituting innovations, and explore new ideas in advance of an actual need [43]. Based on this, research has verified the positive correlation between slack resources and innovation, and it is believed that organizations with rich resources are more inclined to take innovative ideas and behaviors [44]. In fact, the pilot policy is a kind of experiment or demonstration. By adopting the pilot policy, corresponding resources need to be invested in the pilot projects [45]. Therefore, the selection of CSRU pilot counties is more inclined to areas with rich crop straw resources and other auxiliary resources. In 2022, MARA of China intended to select 300 pilot counties and encouraged the selection of pilots from regions with rich crop straw resources, which indicates that straw resource endowment is an important factor influencing the selection of CSRU pilots and policy diffusion. Population size determines the scale of public service demand, which can support the effective implementation of government decisions and the effective utilization of public services [46]. In addition, a greater population size can provide plentiful labor for CSRU promotion. Therefore, the local population size can be taken into consideration in the implementation of the CSRU pilot policy.

Based on above analysis on the effect of resource availability on CSRU pilot adoption, this paper puts forward the following hypotheses:

**H1:**  *In the process of pilot policy diffusion, the availability of crop straw resources within a county can affect its probability of being selected as a CSRU pilot.*

**H2:**  *In the process of pilot policy diffusion, the population size within a county can affect its probability of being selected as a CSRU pilot*.

### 3.2. Internal Capacity

Adaptive capacities within an organization that are associated with financial, organizational, and societal resources play a role in enabling or disabling innovation adaptation [47,48]. Better local economic development can provide more financial support for CSRU promotion. Therefore, regional economic development is seen as a necessary factor in the determination of local government’s adoption of innovative policies [17]. A major difficulty encountered in CSRU practice is the establishment of collection, storage, and transportation system of crop straw. More mechanical capacity can be of great significance to solve this problem and help motivate farmers’ participation in CSRU [49]. Since the pilot selection in China is not a random design, the top decision-makers have specified the details of standard formulation and group definition in the pilot selection. In order to promote CSRU at the local level, the local governments can provide supporting policies to improve local CSRU conditions to keep in accordance with the criteria and standards specified by the top decision-makers [25]. Therefore, the supporting policies from the local government are conducive to improving local CSRU situations and increasing the possibility of being selected as CSRU pilots.

To summarize, the diffusion of CSRU pilot policies needs to consider the internal capacity of the candidates, including local economic development, agricultural mechanical capacity, and local policy supports. Accordingly, the following hypotheses are proposed in this study:

**H3:**  *In the process of pilot policy diffusion, the counties with better economic conditions are more inclined to be selected as CSRU pilots*.

**H4:**  *In the process of policy diffusion, the counties with more agricultural mechanical capacity are more inclined to be selected as CSRU pilots*.

**H5:**  *In the process of policy diffusion, the counties with greater policy supports from local governments are more inclined to be selected as CSRU pilots*.

### 3.3. External Pressure

When adopting new policies, local governments in China are usually affected by the administrative orders and financial relations of the superior governments, and they may also consider the competitive pressure from neighborhood counties [50], which is consistent with the Diffusion of Innovation Theory. By analyzing the previous internal and regional factors influencing policy diffusion, Berry and Berry (1990, 2018) summarized four kinds of policy diffusion mechanisms: learning, competition, public pressure, and mandates [41,51]. Policy diffusion is a learning and referring method based on competitive advantage [20]. The competition mechanism of keeping up with each other is also a common phenomenon in the public policy activities of China [52]. Brown and Cox (1971) proposed the neighborhood effect for diffusion in a spatial context [26]. Specifically, policy diffusion occurs when the policy choices of one jurisdiction (country, state, city, etc.) are affected by the policy choices of other jurisdictions [53]. Therefore, policy choices create externalities for those in the same competition space [54], and this externality is manifested in the diffusion of new policies through the similarity of conditions and shared information in neighboring regions [50]. As agricultural waste, crop straw has displayed specific spatial distribution characteristics due to regional geographical locations and climate conditions [55]. Therefore, counties with similar geographical and climate conditions are more vulnerable to the impact of neighboring governments being selected as CSRU pilots.

In addition to the horizontal diffusion, the pressure and coercion formed in the vertical diffusion, that is, the top-down or bottom-up policy flow, can also impact policy diffusion [56]. Strumpf (2002) stated that a policy pilot is a kind of limited public service [57], which reflects the scarcity of a pilot quota. According to the procedures of pilot selection, the diffusion of the CSRU pilot policy is not driven by the superior government, but more by pressure. The municipal government is responsible for encouraging the counties to apply to be CSRU pilots. In this process, the prefecture level governments are likely to bear the policy pressure from the central and provincial governments. With the in-depth promotion of CSRU at the county level, prefecture level governments are inclined to transfer this pressure to the county level governments. The power relationship between the central government and the township governments in China determines that the higher-level governments play the leading role in promote the diffusion of CSRU pilot policies [52]. Therefore, the counties that confront pressure from the higher authorities may make more efforts in CSRU promotion, resulting in a greater possibility of becoming pilots.

Many studies have shown the importance of public acceptance in renewable energy projects. So public attitudes and opinions also need to be considered in the diffusion of CSRU pilot policies. Relly (2012) found that a favorable environment for news media can have a significant influence on legislation adoption in United Nations member states [58], and this influence is also applicable to the CSRU pilot policy in China. The public opinion and publicity effect can help promote the diffusion of CSRU pilot policies [34,52], especially as the news media plays an important role in setting the policy agenda in China [59]. Therefore, public opinions and attitudes should also be considered in exploring the factors influencing the diffusion of the CSRU pilot policy.

Based on the above analysis on the external pressure, the following hypotheses are proposed:

**H6:**  *In the process of policy diffusion, the counties with greater proximity pressure from the neighboring counties are more inclined to be selected as CSRU pilots*.

**H7:**  *In the process of policy diffusion, the counties with greater pressure from the higher authorities are more inclined to be selected as CSRU pilots*.

**H8:**  *In the process of policy diffusion, the counties with greater pressure from public opinions are more inclined to be selected as CSRU pilots*.

## 4. Data and Methods

By taking 164 counties in Hebei Province as the research sample, this study introduced EHA to establish the methodological model of binary logistic regression, and investigate the effects of various factors on the diffusion of the CSRU pilot policy in China.

### 4.1. Data

Hebei Province, located in North China, with a temperate continental monsoon climate, is a major grain and cotton producing area of China. Hebei Province has a huge amount of crop straw with a high CSRU rate and high willingness to respond to the CSRU pilot policy of the central government. In 2020, the collectable amount of crop straw within this province reached 58.4 million tons, and about 97% of this, namely 56.6 million tons of crop straw, has been utilized as useful resources. By July 2022, more than 30% of the total amount of counties in Hebei Province were selected as CSRU pilots. Therefore, we took Hebei Province as the research area to map the temporal and spatial trace and characteristics in the diffusion of the CSRU pilot policy, and investigate the factors affecting the diffusion and adoption of this pilot policy in China.

For the statistical model, this study used the time-series cross-sectional data at the county level covering CSRU pilot adoption in Hebei province between 2016 and 2020. In order to maintain the integrity of the data, this paper selected 164 counties in Hebei Province with complete data for each year within the observation period. The adoption year of the pilot policy for each county was determined based on the official document of the pilot policy issued by the provincial government, with the adoption year of the local government as the observation end point. According to the EHA method, the year adopted by the local government was taken as the observation end point, and the data of subsequent years were eliminated. Finally, after data screening and sorting, 726 “county-year” observation points were obtained as the data sample of this study.

### 4.2. Methods

This study adopted the logistic regression model and EHA to explore the factors affecting the diffusion and adoption of the CSRU pilot policy. The logistic regression model can help explain why counties were selected as CSRU pilots. However, it could not explain when the counties adopted the pilot policies [51]. To help understand the timing of policy adoption and the diffusion mechanisms, we adopted the EHA, which used a dichotomous dependent variable to describe the adoption of the CSRU pilot policy [60]. EHA is a method that has been widely used in the study of policy innovation diffusion [23,29]. It is capable of explaining and modeling the diffusion effects of the behavior of individuals, and the rules and policies of organizations or governments, and is thus considered an ideal methodology for the study of policy diffusion.

According to EHA, the discrete-time model is usually used when the time unit is a year. The explained variable in the model is the risk rate or occurrence rate, which refers to the probability of a certain event for a specific person at a specific time. However, the occurrence ratio cannot be directly observed, so the binary logistic regression model is generally used for analysis. The model of pilot policy diffusion of CSRU is set as shown in Equation (1) [61]:

(1) $$logic\frac{P_{i,t}}{1-p_{i,t}}=\alpha \beta_{i}\times X_{i}+\varepsilon$$

where, $P_{i,t}$ is the probability that the county will become the pilot of CSRU in year *t*, α is the basic risk rate over time, $\frac{P_{i,t}}{1-p_{i,t}}$ is the odds ratio, and $\beta_{i}$ is the regression coefficient of independent variable $X_{i}$.

### 4.3. Variable Measurement

The dependent and independent variables as well as the relationship model among them is displayed in Figure 3.

(1) Dependent Variable

The dependent variable of the binary logistic regression model is the possibility or probability of a county being selected as a CSRU pilot county or adopting the CSRU pilot policy. According to the discrete-time model of EHA, this is a dummy variable. If county i was selected as the CSRU pilot county in year t, it takes the value of 1 at year t, then the values before year t are all recorded as 0, and the data with respect to this county after year t are all deleted. The dependent variable data come from the documents published by the MARA of China and the Department of Agriculture and Rural Affairs of Hebei Province.

(2) Independent Variable

In terms of resource availability, crop straw resources are measured by straw resource density (SRD), and the population size is measured by population density (PD). The straw resource density of County *i* in year *t*-1 is used to measure the amount of straw resources in year *t*, which is recorded as SRD*_i,t_* [62]. The amount of straw resources here mainly refers to the amount of collectable straw from grain crops. It is calculated by the author according to the grass–grain ratio, collection coefficient of crop straw, and the yields of various grains provided by the MARA of China [63]. Similarly, the population size of County *i* in year *t* is measured by the population density at the end of year *t*-1, which is recorded as PD*_i,t_* [64], and *log*(PD*_i,t_*) is taken to eliminate heteroscedasticity and avoid abnormal data fluctuations.

Among the internal capacity factors, the economic development in Country *i* is measured by the GDP at the end of year *t*-1, which is recorded as GDP*_i,t_*, and *log*(GDP*_i,t_*) is used to eliminate heteroscedasticity and avoid abnormal data fluctuations. The mechanical capacity (MC) is measured by the total power of agricultural machinery and the proportion of grain sown area in County *i*, which is recorded as MC*_i,t_*. The policy support (PS) is measured by the existence of CSRU relevant policies issued by local government of County *i* in year *t*-1, which is recorded as PS*_i,t_*, and it is also a dummy variable. If County *i* has issued CSRU relevant policies in year *t*-1, it will be recorded as 1; if not, it will be recorded as 0. The above data are from the Rural Statistical Yearbook of Hebei Province and the official website of local governments.

Among the external pressure factors, the proximity pressure (PP) of County *i* is measured by the proportion of the number of counties that are selected as CSRU pilot counties among all counties in the prefecture level city in year *t*, which is recorded as PP*_i,t_*, and the proximity pressure of counties directly under the governance of the central government is 0 [34]. The superior government pressure (SGP) of County *i* is measured by the existence of CSRU policies launched by the prefecture level city government in year *t*, which is recorded as SGP*_i,t_*. If the prefecture level cities have issued relevant policies, this variable takes the value of 1, otherwise it is 0. The superior government pressure of the counties directly under the central government is determined based on the existence of CSRU policies issued by the provincial government. The above data come from PKULAW.com. The pressure of public opinions (POP) is measured by the number of CSRU-related reports. The news information and papers about the selected counties were obtained by using COOC V9.94 software to search the database of the China Knowledge Network (CNKI).

Based on the above variable specifications and measurements, Table 2 presents all the variables as well as their measurements and data sources. According to Equation (1), the methodological model of this study is expressed as Equation (2):

(2) $$logic\frac{P_{i,t}}{1-p_{i,t}}=\alpha +\beta_{1}\times {SRD}_{i,t-1}+\beta_{2}\times {PD}_{i,t-1}+\beta_{3}\times {GDP}_{i,t-1}+\beta_{4}\times {MC}_{i,t-1}+\beta_{5}\times {SP}_{i,t-1}+\beta_{6}\times {PP}_{i,t}+\beta_{7}\times {SGP}_{i,t}+\beta_{8}\times {POP}_{i,t}+\varepsilon_{i,t}$$

## 5. Results and Discussions

### 5.1. Temporal and Spatial Characteristics of CSRU Pilot Policy Diffusion

**Figure 4:**
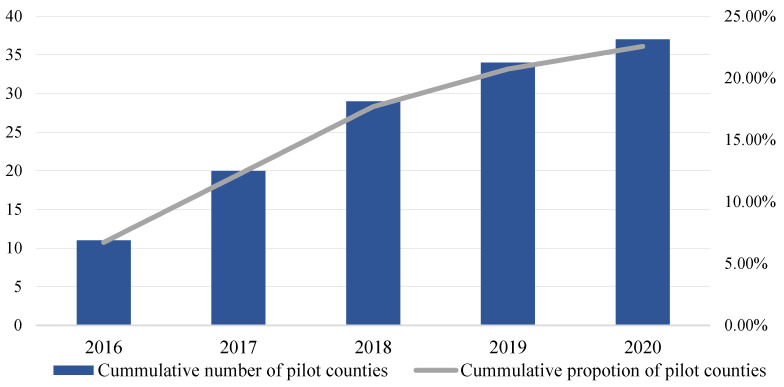
Cumulative number and proportion of pilot counties in Hebei Province, 2019–2020.

In 2016, Hebei Province approved the first batch of eleven CSRU pilot counties, and eleven new pilot counties were added in 2017, and it also actively responded to the CSRU policy requirements of the higher authorities, indicating that Hebei Province has great potential in realizing CSRU. By 2020, there were 37 CSRU pilot counties in this province, as shown in Figure 4, which indicates the fast diffusion of the CSRU pilot policy in this province. Moreover, since it is only seven years since the Chinese government officially started to implement the CSRU pilot policy, it is still at the early rising stage of the S-shaped curve of policy diffusion. With the holistic promotion of CSRU spreading to the whole country, the diffusion of the CSRU pilot policy is expected to be optimistic in the future.

**Figure 5:**
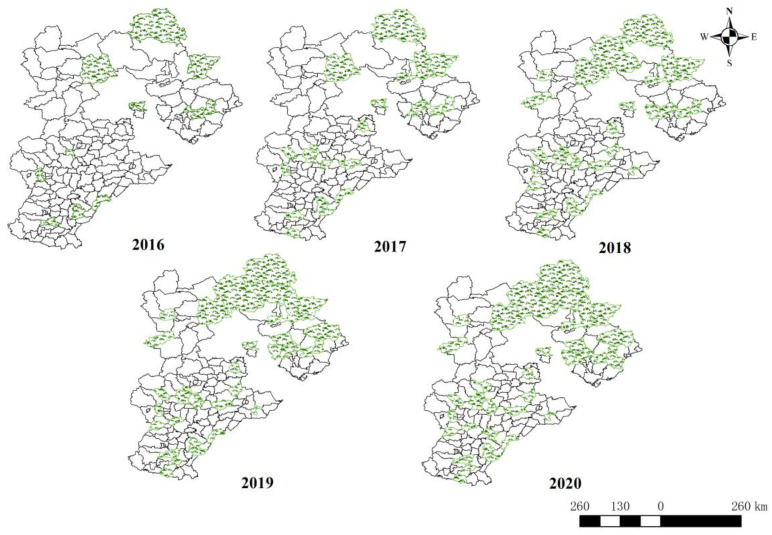
Geographical distribution of CSRU pilot counties in Hebei Province from 2016 to 2020.

To vividly reflect the spatial distribution characteristics of the CSRU pilot policy diffusion, this study presented the diffusion process of the CSRU pilot policy in Hebei Province by using ArcGIS V10.8, a geographic information system software. The results are as shown in Figure 5, and some characteristics of CSRU pilot diffusions can be identified.

Firstly, the overall distribution of the CSRU pilot counties in Hebei Province is characterized by the evolution from dispersion to aggregation, as more and more pilot counties have been selected for the pilot list. Secondly, the aggregation areas are mainly concentrated in the northeast and southwest of the province, and have gradually spread to the middle counties from 2020. The counties in the northeast of Hebei Province have shown more obvious aggregative effect than those in the southwest and middle of the province. For instance, the proportion of pilot counties in Chengde City and Qinhuangdao City reached 45% and 42%, respectively. Thirdly, more obvious diffusion effects of the CSRU pilot policy can be observed within the same provincial city, as most provincial cities have at least one county selected as a pilot every year.

### 5.2. Descriptive Statistics and Collinearity Analysis

The descriptive statistical information of the data sample is shown in Table 3. In order to detect the existence of collinearity among the variables, this paper conducted a collinearity test, and the results are also shown in Table 3. The variance expansion factor (VIF) is a commonly used indicator for detecting collinearity, and the VIF values with respect to all independent variables are less than 10, indicating no serious collinearity among the independent variables [65].

### 5.3. Results of Regression Analysis

By using SPSS V26.0, binary logistics regression analysis was conducted. The results of the Omnibus Test (Table 4) indicates that explanatory variables in this model have well explained the variance of the outcome variables at the significance of 0.000, and the model summary (Table 5) also indicates the good fitting effect of the model. Table 6 indicates that the model explains approximately 95.2% of the variance in the event of adopting a pilot county.

The results of binary logistics regression analysis are presented in Table 7. Among the independent variables, straw resource density (SRD), population density (PD), policy support of local government (PS), and proximity pressure from neighboring counties (PP) are proved to have significant effects on the diffusion of the CSRU pilot policy in Hebei Province, while the effects of the rest of the factors on the diffusion of CSRU pilot policies fail to be supported by the empirical analysis, as shown in Table 8.

In terms of resource availability, straw resource density (SRD) has an obvious positive effect on the diffusion of the CSRU pilot policy at the significance level of 5%, so H1 is supported by the empirical results. Crop straw is an important biomass resource. When the straw resource density within a county increase by one ton/ha, the possibility of being selected as a pilot increases by 23.2% (odds ratio = 1.232), which indicates that the counties with abundant crop straw resources are more likely to be selected as CSRU pilot counties. In fact, the supply of crop straw plays an important role in the large-scale utilization of crop straw [66], while a big problem in utilizing crop straw as a material for energy production is the shortage of raw materials [63]. Due to the large amount of crop straw resources, 1253 counties and districts were selected as CSRU pilots in 2022. It can be expected that priority will still be given to those areas with rich crop straw resources in the selection of CSRU pilots in the future.

Population density (PD) has been proved to have a negative impact on the adoption of the CSRU pilot policy at the significance level of 5%, which means H2 is supported by the empirical results, and counties with smaller population densities are more conducive to being selected as CSRU pilots. Presently, CSRU in China is mainly realized by returning crop straw back to the fields as fertilizer and utilizing it as material for fodder production, especially in Huang-Huai-Hai Plain, which covers most of the territory of Hebei Province, and is one of the main distribution areas of cultivated land in China. Especially, the northern counties in Hebei Province have lower population densities than the counties in the south, while more CSRU pilot counties seems to be selected in the north. A low population density indicates that the agricultural land area per capita is large and the labor force is relatively insufficient, so the farmers are more inclined to return the crop straw directly to the field or sell it to factories that take crop straw as a raw material [62]. Therefore, a lower population density is more conducive to the implementation of CSRU in rural areas.

Among the international capacity factors, policy support (PS) has shown a positive impact on the diffusion of the CSRU pilot policy at the significance level of 1%, which means H5 has been supported by the empirical results. Policy supports from local government can increase the probability of the county being selected as a pilot by nearly ten (odds ratio = 10.702), which indicates the great effects of the willingness and policy support of the county government in the selection of CSRU pilots. By contrast, H3 and H4 have failed to be supported by the empirical results. The effect of economic development (GDP) on the diffusion of the CSRU pilot policy is not significant. The possible reason could be that, at the early stage of CSRU promotion, it mainly relies on the policy and financial support from local government, and the economic benefits from CSRU are not enough to attract enterprises and investors to participate. The mechanical capacity (MC) is also proved to have no significant influence on the diffusion of the CSRU pilot policy, which is very likely to be induced by the fact that CSRU has gradually been separated from agriculture, and agricultural mechanical capacity would not generate any impacts on CSRU promotion.

Among the external pressure factors, the proximity pressure (PP) from neighboring counties has a positive impact on the diffusion of the CSRU pilot policy at the significance level of 1%, which means H6 has been supported by the empirical results, indicating that a county being selecting as a CSRU pilot can stimulate the other counties in a prefecture level city in CSRU promotion. It also shows that the innovation diffusion mechanism in neighboring areas plays an important role in the diffusion of the CSRU pilot policy. Meanwhile, the effects of the pressures from superior government (SGP) and public opinions (POP) on the diffusion of the CSRU pilot policy are proved to be not significant, which indicates that H7 and H8 are not supported by the empirical results. Thus, the pressure from superior government and public opinions has no impact on the pilot selection of a county. The main reason could be that China’s CSRU pilot policy is more like an incentive policy rather than a punitive policy, so both the pressures from superior government and public opinions could not affect the selection of CSRU pilots.

### 5.4. Discussion

There are also some other studies that have investigated the factors affecting CSRU. For instance, Li et al. (2022) summarized the factors influencing farmers’ willingness and behavior in CSRU, which included individual and household characteristics, resource endowment, cognitive factors, and other endogenous factors [37]; By conducting content-analysis-based data collected from semi-structured interviews and official publications, Del Valle and Jiang (2022) found that Chinese farmers’ adoption of CSRU was mainly affected by individual characteristics, farmland and climate conditions, crop types, costs, government plans and supporting policies, and social networks [38]. Despite the fact that many factors influencing CSRU adoption have been summarized, their effects failed to be validated through quantitative analysis. Based on the Unified Theory of Acceptance and Use of Technology and quantitative analysis, He et al. (2018) found that, in addition to factors such as time cost, economic cost, and influence from other persons, the interpersonal trust and institutional trust also affected farmers’ willingness in CSRU [35]. However, these factors are mainly determined from the perspective of farmers and rural households. By contrast, this study identified the factors affecting CSRU policy diffusion from the aspects of resource availability, internal capacity, and external pressure, and validated their effects through the binary logistic regression model.

The societal benefits of this study lie in the following aspects: firstly, it summarizes the factors affecting the diffusion of the CSRU policy from the aspects of resource availability, internal capacity, and external pressure, which provides a framework for determining the critical factors in policy diffusion studies. Secondly, it identifies the important factors that can affect the diffusion of the CSRU policy in China, which can provide useful implications to policy makers in the utilization of crop straw.

## 6. Conclusions and Policy Implications

By taking Hebei Province as an example, this study mapped the temporal and spatial characteristics of the diffusion of the CSRU pilot policy among the counties in this province, and conducted an EHA by establishing a binary logistic regression model to identify the specific factors that determine the diffusion of the CSRU pilot policy in China. The conclusions can be drawn as follows:

(1) The CSRU pilot policy diffuses rapidly in Hebei Province, and a gradual diffusion from the northeast and southwest to the other parts has been presented. As a pilot policy of replacing subsidies with awards, the CSRU pilot quota is a kind of scare resource. However, about one fourth of the total number of counties in Hebei Province have been selected as CSRU pilot counties within the last five years, indicating the fast diffusion of this pilot policy in this province, considering this diffusion process is still at the initial rising stage of the S-shaped curve. In addition, a trend of gradual diffusion from the northeast and southwest to the central parts is also displayed, and great competition in pilot selecting can be witnessed among different prefecture level cities.
(2) Resource availability, namely straw resource density and population density, are proved to have significant effects on the diffusion of the CSRU pilot policy. To be specific, straw resource density has a positive effect on the diffusion of the CSRU pilot policy, as the abundant straw resources provide the physical basis for CSRU promotion. Population density has shown to have a negative effect on the diffusion of the CSRU pilot policy. A lower population density means more arable land per capita, which is more conducive to the utilization of crop straw, and this can increase the possibility of being selected as a CSRU pilot.
(3) Among the internal capacity factors, the policy support of local government is conducive to the diffusion of pilot policies. The counties with favorable and supporting CSRU policies indicates the willingness and actions of local government in CSRU promotion, which can attract enterprises and farmers to participate in CSRU and form a good industrial basis for the large-scale utilization of crop straw. By doing this, it can help the local government to win the reward of being selected as a CSRU pilot and receive more financial subsidies to promote the further development of CSRU.
(4) The proximity pressure from neighboring counties can have a positive effect on the diffusion of the CSRU pilot policy. Due to the similar geographical and climate conditions within a region, a county being selected as a CSRU pilot can generate pressure on other counties within this region that have failed to be selected as pilots. This pressure will push them to improve their CSRU performance, which then increases the possibility of them being selected as CSRU pilots.

To promote CSRU in China, the following policy implications are provided:

Firstly, in addition to considering the availability of crop straw, the attitude of stakeholders, especially the willingness of local governments, can play a vital role in CSRU promotion. In fact, CSRU promotion led by the government is more conducive to the participation of other stakeholders and the creation of a favorable market that can facilitate CSRU. Therefore, the CSRU pilot policy in China gives full play to the role of the county government. These three elements (local government, enterprises and farmers) are indispensable in CSRU promotion, and they form a solid three-element diffusion mechanism.

Secondly, it is important to collect information such as the availability of crop straw and the attitudes and supporting policy of local government, which is the foundation for the further diffusion of the CSRU pilot policy. In recent years, the MARA of China has gradually strengthened the establishment of crop straw accounts (including data on CSRU rates and the amount of crop straw that has been utilized by different technological pathways in each county, which have not yet been released to the public). At the same time, it is more important to implement supporting policies to promote CSRU by local government, so as to increase the probability of success in CSRU pilot selection and obtain the pilot subsidies to establish the industrial chain for realizing CSRU and activate the CSRU market.

In addition, a complete industrial chain of straw resource utilization needs to be established. Actually, the ultimate goal of “promoting CSRU in the whole county” is to form an independent and complete market industrial chain. The industrial chain will not be limited to a specific county, but will attract the stakeholders in neighboring counties. Therefore, local county governments should use this opportunity to attract straw utilization companies, and stimulate the willingness of farmers and self-employed households to establish a complete industrial chain for realizing CSRU, and maximize economic, social, and environmental benefits.

## Figures and Tables

**Table 1:** Supporting policies and efforts in CSRU pilot promotion in China since 2009.

Year	Policy Contents
2009	The MARA of China launched 16 large- and medium-sized pilot biogas projects with crop straw as the base material in 14 provinces.
2011	The NDRC, MARA and MOF of China issued the Implementation Plan for CSRU during the 12th Five Year Plan, which proposed the implement of the CSRU pilot policy and technology. In 2016, the MARA and MOF issued the Notice on Carrying out CSRU Pilot Policy to Promote the Improvement of Cultivated Land Quality, and invested CNY one billion to select ten provinces and autonomous regions as pilots for practicing CSRU.
2017	The Opinions on Innovating Systems and Mechanisms to Promote Green Agricultural Development was issued, then the Straw Treatment Action in Northeast China was launched, which aims to promote CSRU in 60 major corn-producing counties in Northeast China, and requires the whole county to promote the full quantitative utilization of crop straw.
2018	The central government allocated CNY 1.3 billion of financial funds to support 143 pilot counties in practicing CSRU.
2019	The MARA decided to promote CSRU comprehensively, and required the selection of a number of counties (including districts and county level cities) with rich straw resources and great potential to promote CSRU throughout the county.
2020	CNY 2.58 billion was allocated by the central government to support 157 CSRU pilot counties in Northeast China to promote CSRU.
2021	Another CNY 2.7 billion was invested by the central government to promote CSRU around the country.
2022	The MARA intended to carry out special deployment for CSRU promotion, take fertilizer, fodder, and energy biomass as the main target products of CSRU, and promote coordinated development at both the demand and supply sides. In addition, it planned to select 300 pilot counties and 600 demonstration bases of CSRU around the country.

**Table 2:** Variable measurements and data sources.

Variable Type		Indicators	Measurement	Data Source
Dependent variable		Adoption of CSRU pilot policy	If being selected as pilot county, it takes the value of 1, otherwise takes 0.	DARA of Hebei Province
Independent variable	Resource availability	Straw resource density (SRD)	Collectable amount of straw/planting area of grain crops (t/ha)	Hebei Rural Statistical Yearbook
		Population density (PD)	Total population/planting area of grain crops (ha per capita)	Hebei Rural Statistical Yearbook
	Internal capacity	Economic development	GDP (CNY per capita)	Hebei Rural Statistical Yearbook
		Mechanical capacity (MC)	Total power of agricultural machinery/planting area of grain crops (kW/ha)	Hebei Rural Statistical Yearbook
		Policy support (PS)	If local government has issued CSRU policies, it takes the value of 1, otherwise it takes 0.	Official website of local governments
	External pressure	Proximity pressure (PP)	Number of CSRU pilot counties/total number of counties within the city.	DARA of Hebei Province
		Superior government pressure (SGP)	If the superior government has issued CSRU related policy, it takes the value of 1, otherwise it takes 0.	PKULAW.com
		Public opinion pressure (POP)	Number of news and journal papers on CSRU in the county.	CNKI

**Table 3:** Descriptive statistical analysis results.

Variable	Observations	Mean	Standard Deviation	MIN	MAX	VIF
SRD	726	7.31	1.84	0.64	14.75	1.060
PD	726	1.20	0.56	0.18	4.20	1.226
GDP	726	6.14	0.31	5.25	6.98	1.144
MC	726	16.62	16.45	1.87	236.72	1.199
PS	726	0.25	0.44	0.00	1.00	1.024
PP	726	0.04	0.04	0.00	0.22	1.027
SGP	726	0.79	0.41	0.00	1.00	1.083
POP	726	0.07	0.25	0.00	2.00	1.007

**Table 4:** Omnibus tests of model coefficients.

		Chi-Square	Degree of Freedom	Significance
Step 1	Step	84.879	8	0.000
	Block	84.879	8	0.000
	Model	84.879	8	0.000

**Table 5:** Model summary.

−2 Log Likelihood	Cox & Snell R^2^	Nagelkerke R^2^
201.595	0.110	0.338

**Table 6:** Classification Table.

	Observed		Predicted		
			Adoption		Percentage Correct
			Not Adopted	Adopted	
Step 1	Adoption	Not adopted	687	3	99.6
		Adopted	32	4	11.1
	Overall Percentage				95.2

**Table 7:** Results of binary logistic regression analysis on the diffusion of the CSRU pilot policy in the counties of Hebei Province.

Variable	Regression Coefficient	Standard Error	Odds Ratio	*p*-Value
SRD	0.209	0.099	1.232	0.036 **
PD	−2.258	0.915	0.105	0.014 **
GDP	0.223	0.716	1.250	0.756
MC	−0.031	0.028	0.970	0.271
SP	2.370	0.432	10.702	0.000 ***
PP	24.079	4.697	2.86 × 10^10^	0.000 ***
SGP	0.035	0.506	1.036	0.944
POP	0.906	0.648	2.475	0.162
Constant	−5.846	4.258	0.003	0.175

Note: *** means significant at the level of 1%; ** means significant at the level of 5%; * means significant at the level of 10%.

**Table 8:** Results of hypothesis verification.

Hypotheses	Results
H1: In the process of pilot policy diffusion, the availability of crop straw resources within a county can affect its probability of being selected as a CSRU pilot.	Supported
H2: In the process of pilot policy diffusion, the population size within a county can affect its probability of being selected as a CSRU pilot.	Supported
H3: In the process of pilot policy diffusion, the counties with better economic conditions are more inclined to be selected as CSRU pilots.	Not supported
H4: In the process of policy diffusion, the counties with more agricultural mechanical capacity are more inclined to be selected as CSRU pilots.	Not supported
H5: In the process of policy diffusion, the counties with greater policy supports from local governments are more inclined to be selected as CSRU pilots.	Supported
H6: In the process of policy diffusion, counties with greater proximity pressure from the neighboring counties are more inclined to be selected as CSRU pilots.	Supported
H7: In the process of policy diffusion, the counties with greater pressure from the higher authorities are more inclined to be selected as CSRU pilots.	Not supported
H8: In the process of policy diffusion, the counties with greater pressure from public opinions are more inclined to be selected as CSRU pilots.	Not supported

## Data Availability

The data presented in this study are available upon request from the corresponding author.
